# *In situ* synthesis and electronic transport of the carbon-coated Ag@C/MWCNT nanocomposite

**DOI:** 10.1039/c8ra00078f

**Published:** 2018-02-15

**Authors:** Dongxing Wang, Da Li, Javid Muhammad, Yuanliang Zhou, Ziming Wang, Sansan Lu, Xinglong Dong, Zhidong Zhang

**Affiliations:** Key Laboratory of Materials Modification by Laser, Ion and Electron Beams (Ministry of Education), School of Materials Science and Engineering, Dalian University of Technology Dalian 116023 China dongxl@dlut.edu.cn +86-411-84706130; Shenyang National Laboratory for Materials Science, Institute of Metal Research, International Center for Materials Physics, Chinese Academy of Sciences 72 Wenhua Road Shenyang 110016 People's Republic of China zdzhang@imr.ac.cn +86-24-23971859

## Abstract

A nanocomposite of Ag@C nanocapsules dispersed in a multi-walled carbon nanotube (MWCNT) matrix was fabricated *in situ* by a facile arc-discharge plasma approach, using bulk Ag as the raw target and methane gas as the carbon source. It was found that the Ag@C nanocapsules were ∼10 nm in mean diameter, and the MWCNTs had 17–32 graphite layers in the wall with a thickness of 7–10 nm, while a small quantity of spherical carbon cages (giant fullerenes) were also involved with approximately 20–30 layers of the graphite shell. Typical dielectric behavior was dominant in the electronic transport of Ag@C/MWCNT nanocomposites; however, this was greatly modified by metallic Ag cores with respect to pure MWCNTs. A temperature-dependent resistance and *I*–*V* relationship provided evidence of a transition from Mott–David variable range hopping [ln *ρ*(*T*) ∼ *T*^−1/4^] to Shklovskii–Efros variable range hopping [ln *ρ*(*T*) ∼ *T*^−1/2^] at 5.4 K. A Coulomb gap, *Δ*_C_ ≈ 0.05 meV, was obtained for the Ag@C/MWCNT nanocomposite system.

## Abstract 2

An electric transition from ln *ρ*(*T*) ∼ *T*^−1/4^ to ln *ρ*(*T*) ∼ *T*^−1/2^ hopping conduction happened at 5.4 K *in situ* synthesis of Ag@C/MWCNTs nanocomposite.

## Graphical abstract

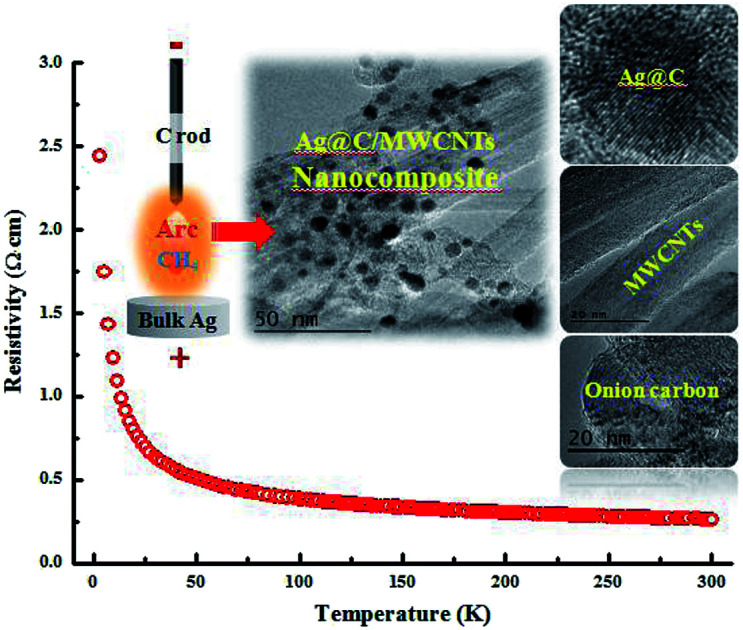

## Introduction

In recent years, carbon nanotubes (CNTs) have attracted significant research efforts, because of their unique structures, special properties, and tremendous potential applications in the fields of composite materials, electrode materials, field emitters, nanoelectronics, and nanosensors, for example.^1–3^ It was found that the transport behavior of the carbonaceous species depends on the morphology and structural perfection of the CNT; *e.g.* single-walled carbon nanotubes (SWCNTs) or their bundles show classical transport properties such as Coulomb blockade, level quantization, Luttinger liquid characteristics, and ballistic transport, for example.^4–6^ In contrast, in multi-walled carbon nanotubes (MWCNTs), it is usually difficult to make electrical contacts between the inner carbon layers, so the total conductance would be significantly limited by the charge carrier transport.^7^ Variable range hopping (VRH) conduction, weak localization, resonant tunneling phenomena, universal conductance fluctuations, or Aharonov–Bohm oscillations of magnetoresistance may appear and become dominant in the electronic transport behaviors of MWCNT-containing systems.^8–11^ As typical carbonic nanostructures, the carbon-fullerene family has been extensively investigated and show fundamental importance in the advancement of science and engineering as a consequence of their unique electronic and magnetic properties.^12,13^ To meet increasingly high requirements for applications, the fabrication of composites through the integration of CNTs with complementary metal nanoparticles has proved an effective route to gain excellent performances.^14^

Silver is a typical metal with a low melting point, high electrical conductivity, electrochemical activity, and favorable specialties. In recent years, nanocomposites of Ag nanoparticles combined with carbon nanotubes (Ag/CNTs) have been synthesized and applied in many fields. For instance, Melvin *et al.* evaluated the electromagnetic wave absorption properties of single/double-layer Ag/CNTs nanocomposites.^15^ Ma *et al.* reported highly conductive flexible adhesives (CFAs) composed of nanoscale Ag flakes, CNTs and nitrile butadiene rubber.^16^ Pillai *et al.* reported a conductive hybrid network consisted of silver nanowires (Ag NWs) and SWCNT, which has been demonstrated potential in high energy density flexible-solid-state supercapacitor.^17^ Dong *et al.* fabricated the Ag/CNTs networks for a highly conductive film.^18^ Li *et al.* theoretically investigated the atomic and electronic structures of silver-filled SWCNTs by using first-principles calculations.^19^ Feng *et al.* revealed that the electrical resistivity of Ag/CNT composite increases slightly with the volume fraction of CNTs in a volume range below 10 vol%.^20^ Luo *et al.* synthesized Ag/CNT sorbent for the removal of elemental mercury from flue gases.^21^ Jung *et al.* also prepared airborne Ag/CNT hybrid nanoparticles, for use as the antimicrobial in air filtration.^22^ In the above studies, almost all Ag/CNT composite systems were fabricated by mechanically mixing CNT powders and Ag nanoparticles in solvents, while a few were obtained *via* an aerosol approach or a chemical reaction method.^22,23^ The most interesting research has concerned the electrical properties of Ag/CNT nanocomposites, which depend on the intrinsic capabilities of the individual parent, and also on their morphologies and interfacial characteristics. However, the electronic transport behaviors have rarely been examined to date.

To date, the fabrication of Ag/CNT nanocomposites has been achieved through a large number of strategies. To the best of our knowledge, the process of arc-discharge plasma has not been reported for the synthesis of such multicomponent composites. Here, this facile method was first applied in a one-step synthesis of the composite with carbon-coated Ag nanocapsules (Ag@C) embedded in a MWCNT matrix. Arc-discharge plasma is a conventional method with the merits of easy operation, controllable preparation conditions, large yield of nanopowders, and less pollution in the environment. Investigation of the electronic transport behaviors of Ag@C/MWCNT composites were also carried out, which helps to gain an insight into the influence of Ag@C nanocapsules on the electronic performance of the assembled MWCNTs. A testing sheet for as-prepared Ag@C/MWCNT powders was prepared using a mechanical press without any binder, to insure its high purity and well-defined junctions among the nanoparticles and MWCNTs. Electronic transport performances have been measured in the temperature range from 2 K to room temperature.

## Experimental details

### Synthesis of Ag@C/MWCNT nanocomposite

Ag@C/MWCNT nanocomposite powders were prepared using arc-discharge plasma, as reported in our previous publication (Dong *et al.*).^24^ The raw target was pure bulk Ag (99.99% in purity), which served as the anode for arc-discharge to be evaporated. A carbon rod was used as the opposite cathode. After evacuating the chamber to 1.0 × 10^−2^ Pa, it was filled with methane gas to 1.0 × 10^−2^ MPa. After ignition of the arc, the voltage was maintained at about 20 V, depending on the gap between the two electrodes, and the current was set at 100 A. Following a series of manipulations (*i.e.*, evaporation of Ag target, nucleation and growth of fine clusters and passivation for over 12 hours), the powder product was collected from the water-cooled wall of the work chamber.

### Structural characterization and resistivity measurement

Crystal phases of as-prepared Ag@C/MWCNT powders were confirmed by X-ray diffraction (XRD; PANalytical Empyrean) using Cu Kα radiation (*λ* = 1.5416 Å). Morphologies, interfaces and crystallographic details were characterized by scanning transmission electron microscopy (STEM; NOVA NanoSEM 450, 300 keV). Raman spectra were recorded using a laser excitation wavelength of 632.8 nm. X-ray photoelectron spectroscopy (XPS; Thermo Escalab 250Xi) utilizing monochromatic Al Kα (*hv* = 1486.6 eV) radiation as the theta probe was adopted to analyze the surface species. For the resistivity measurements, the testing sample was prepared by pressing the as-prepared nanopowders into a slice under an axial pressure of 20 MPa in a steel die, and cutting to a size of 10 × 10 mm. The temperature dependence of resistivity and the *I*–*V* curve were measured on a physical property measurement system (PPMS; Quantum Design).

## Results and discussion

### Structure, morphology and surface species of Ag@C/MWCNTs

**Fig. 1:**
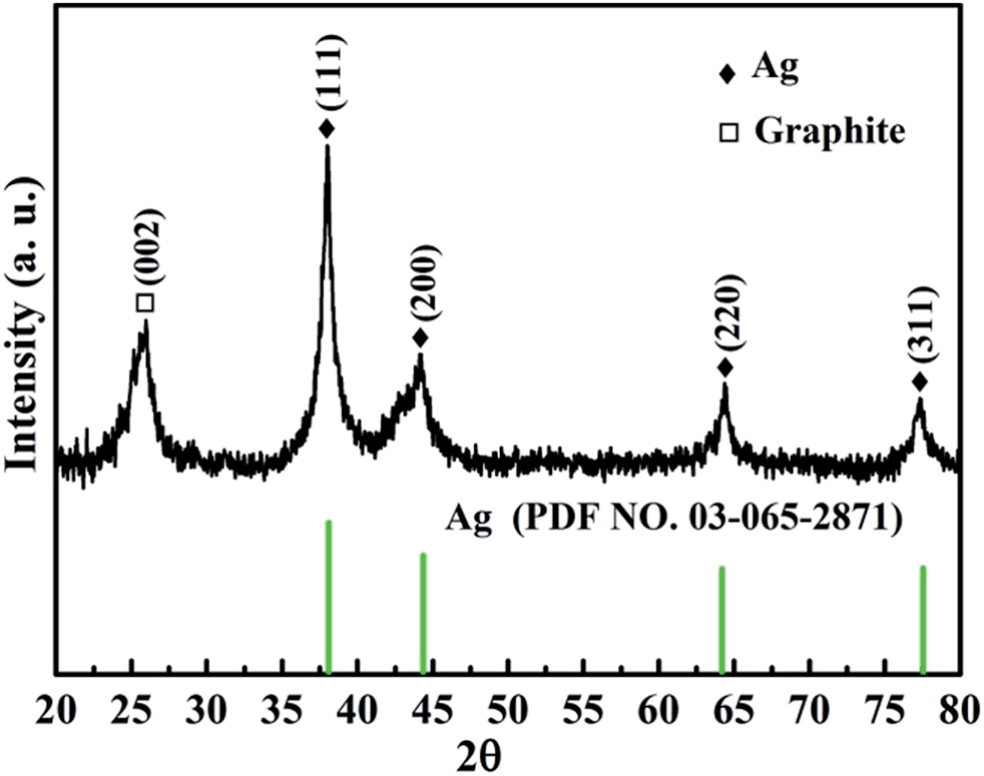
XRD pattern of the as-prepared Ag@C/MWCNT composite powder.

**Fig. 2:**
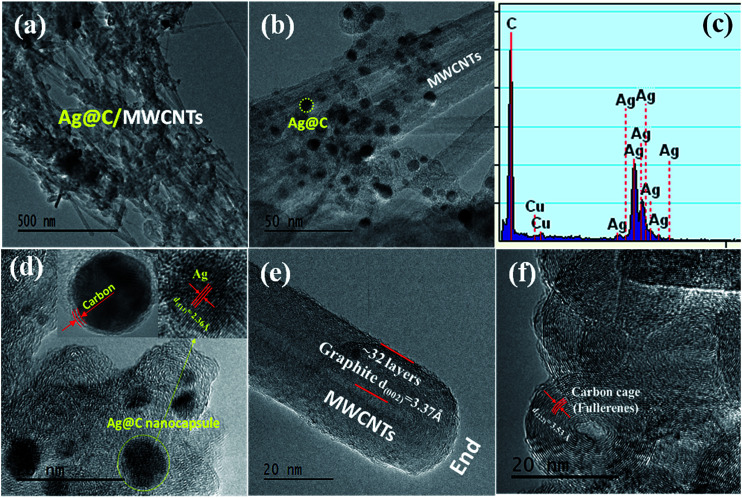
HRTEM images and EDX spectrum of Ag@C/MWCNT composite powder. (a, b) Full views; (c) EDX spectrum; (d) Ag@C nanocapsule; (e) typical MWCNT; and (f) hollow carbon cages with an onion-like structure.

**Fig. 3:**
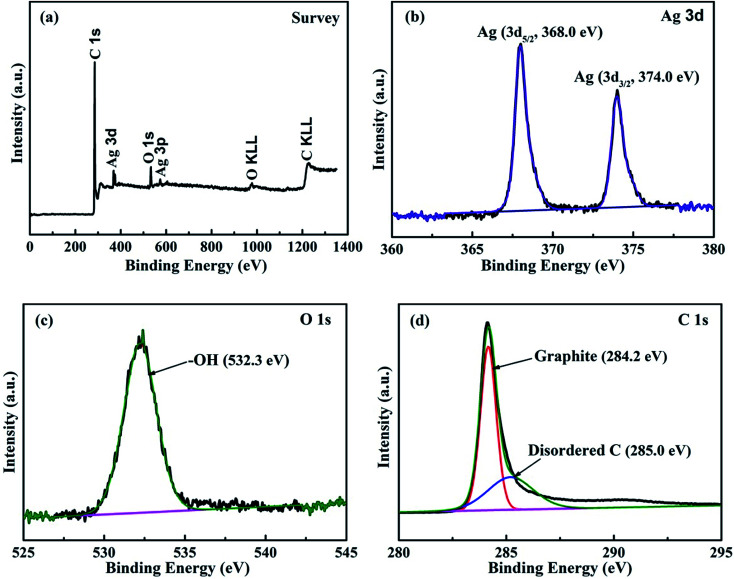
XPS spectra of Ag@C/MWCNT composite powder. (a) Survey spectra; (b) binding energies of Ag 3d electrons; (c) binding energies of O 1s electrons; (d) binding energies of C 1s electrons.

Fig. 1 shows the XRD pattern of the as-prepared Ag@C/MWCNT composite powder. The characteristic peaks at 2*θ* = 38.0°, 44.2°, 64.4°, and 77.3° are the diffractions from the crystallographic planes (111), (200), (220), and (311) of the metallic Ag phase (PDF no. 00-004-0783), while the peak at 2*θ* = 26.1° corresponds to the (002) plane of the graphite phase (PDF no. 00-001-0646). The detected graphite phase was thought to be from the MWCNTs and onion carbon structures existing in the powder; both will be further confirmed by the following high-resolution transmission electron microscopy (HRTEM) images (see Fig. 2). No silver oxide is visible in the XRD pattern and XPS measurement (see Fig. 3), for the reason that the metal Ag particles are protected by carbonaceous layers on the surface. It is the carbon-coated Ag@C nanocapsules (NCs) that are present.

The morphologies and structures of Ag@C/MWCNT powders are observed as shown in HRTEM images of Fig. 2(a)–(f) under different magnifications. Fig. 2(a) and (b) show the full view of Ag@C/MWCNT powders; spherical Ag@C NCs, one-dimensional MWCNTs and some carbon cages are found. To further confirm the composition of the powder, the energy dispersive X-ray (EDX) spectrum was recorded, as shown in Fig. 2(c). It is seen that the Ag@C/MWCNT composite consists of Ag (31 wt%) and C (69 wt%) elements, while a small quantity of carbon and the Cu element come from the sample holder used for the TEM observation. The mean size of the spherical Ag@C NCs is ∼10 nm in diameter, and the Ag core is further confirmed by the detailed lattice image (inset of Fig. 2(d)), in which the single crystal Ag core is demonstrated by the lattice spacing of 0.236 nm matching to the distance of (111) plane in fcc-Ag. It is also seen in the inset of Fig. 2(d) that the Ag nanoparticle is covered with a disordered carbon shell, 2–3 nm in thickness, or embedded in the amorphous carbon matrix. Such carbon coating of Ag@C NCs has an effective ability to protect the active Ag core against oxidation, and is also the reason that no silver oxide is detected in the XRD pattern (Fig. 1) and XPS analysis (see Fig. 3(b)). A typical MWCNT, as displayed in Fig. 2(e), is mature, closed, and perfect in its structure, with ∼32 graphite layers and an outside diameter of ∼30 nm. It is obvious that MWCNTs have a highly oriented structure comprising of numerous parallel graphitic layers, retaining structural continuity even to the end of the CNT. As shown in Fig. 2(f), a large number of spherical carbon cages (giant fullerenes) are found with approximately 25–30 graphite layers in shells, verified from the interplanar spacing of 3.56 Å belonging to (222) lattice planes of the fullerenes (PDF no. 00-049-1717). The existence of hollow cages is considered to result from the migration of the metallic Ag core out of the original cage in the solidification process.

**Fig. 4:**
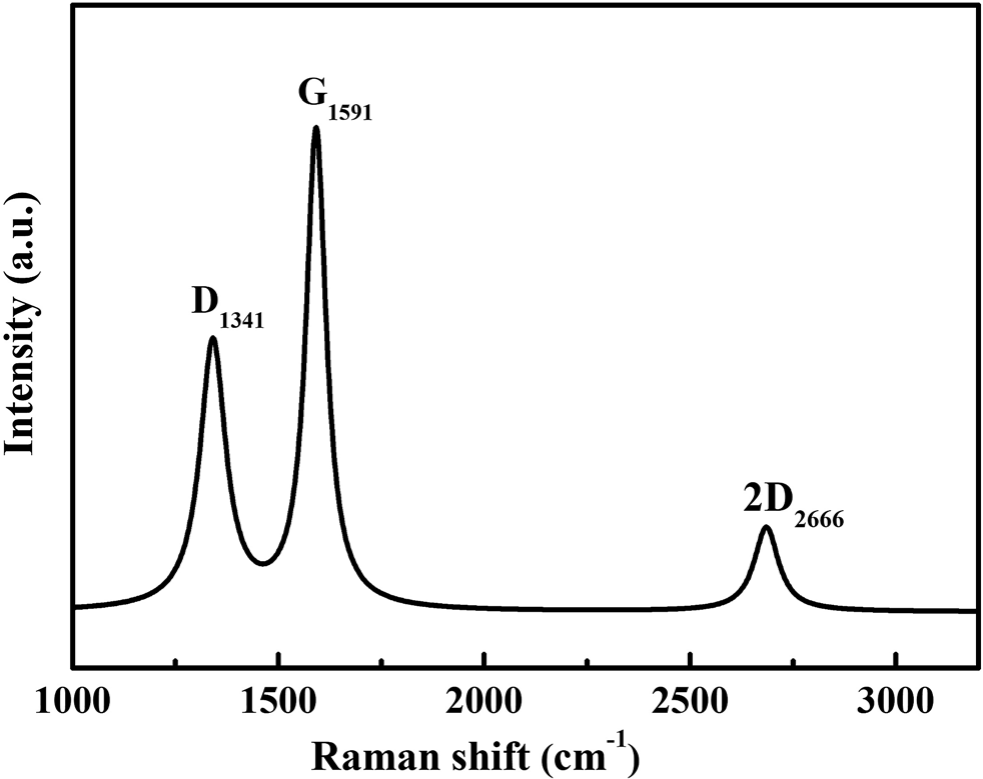
Raman spectrum of the Ag@C/MWCNT composite powder.

In order to identify the chemical composition of the Ag@C/MWCNT composite powder, the XPS analysis was carried out as shown in Fig. 3. The survey spectra of Fig. 3(a) indicate the emissions from the Ag, C, and O elements, and the contents may be calculated as 6.61 wt% of Ag, 86.93 wt% of C, and 6.41 wt% of O. The oxygen is attributed to the adsorbed –OH/O_2_ species in the air, the carbon arises from the MWCNTs, onion-like carbon cages, and disordered carbon nanostructures, while the Ag exists in the capsulated core–shell structures. Fig. 3(b)–(d) show the detailed photoelectron spectra of Ag, O, and C elements, respectively. The peaks in Fig. 3(b) confirm pure Ag with zero valances through the binding energies of Ag 3d electrons (368.0 eV for 3d_5/2_, 374.0 eV for 3d_3/2_ electrons),^25^ without any silver oxides, which indicates that the pure Ag has been protected by the carbonaceous species. The feature peak of O 1s electrons at a binding energy of 532.26 eV is attributed to the adsorbed –OH/O_2_ species in air (Fig. 3(c)), while the absence of any peaks in the range 528–530 eV (the binding energies for Ag–O) suggests no silver oxide passivated in the composite. Fig. 4(d) reveals the detailed spectrum of the C element, with the peak of C 1s electrons at binding energies of 284.22 eV and 285.0 eV corresponding to the sp^2^-hybridized graphite-like carbon atoms of MWCNTs and the sp^3^-hybridized carbon atoms in a disordered structure, respectively.^26^

The graphitization degree of the Ag@C/MWCNT composite powder was analyzed by Raman spectra, as shown in Fig. 4. Two intensive peaks denoted as D-band at 1341 cm^−1^ and G-band at 1591 cm^−1^ correspond to the A_1g_ and E_2g_ of carbon vibration modes, respectively. The D-band suggests a disordered feature of the carbon structure and the G-band indicates perfect graphite. Both peaks are related to the sp^2^ electronic configuration containing electrons in the π orbital and appear in the majority of Raman spectra of graphitic materials of a small crystallite size (so-called semicrystalline graphite). The D-peak, normally assigned to the presence of in-plane substitution heteroatoms, vacancies, grain boundaries, or other defects, indicates here a large amount of disordered carbon existing in the Ag@C/MWCNT powders, particularly in MWCNTs, onion-like carbon cages, and the disordered carbon structures, as observed in the HRTEM images of Fig. 2. A strong G-band is typical for graphite or carbon blacks, and originates from the stretching vibration of any C–C pairs at sp^2^ sites,^27^ and represents here a high crystallinity and mature degree of MWCNTs and carbon cages. The intensity ratio of *I*_D_/*I*_G_ gives an intuition into the degree of carbonic crystallinity; here it is approximately 0.63 as obtained from a Lorentz function fitting on the Raman spectrum, which is smaller than 0.93 of pure MWCNTs.^28^ The value of *I*_D_/*I*_G_ suggests that the graphitization degree in the Ag@C/MWCNT composite is relatively high, due to the high energy conditions of the arc-discharge plasma. Additionally, the peak denoted as 2D-band at around 2666 cm^−1^ is thought to originate from the finite crystal size of graphite, which is an overtone of the D-peak in the case of a total lack of *c*-axis order.

### Formation mechanism of one-step synthesized Ag@C/MWCNT powder

**Fig. 5:**
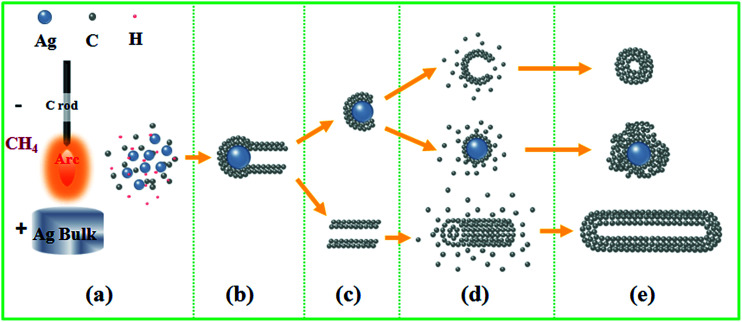
Schematic formation of Ag@C/MWCNT composite powder by *in situ* arc-discharge plasma. (a) Gasification of the Ag bulk; (b) absorption growth of C atoms; (c) separation of MWCNTs; (d) second absorption of C atoms; (e) formation of CNTs, Ag@C, carbon cages.

A schematic diagram for the *in situ* formation of Ag@C/MWCNT composite powder by arc-discharge plasma is shown in Fig. 5. The plasma is a heat source which evaporates the bulk target into the gaseous state, then follows a series of nucleation, growth, diffusion, and passivation processes. The temperature of the arc flame may reach higher than 10 000 K, depending on the atmospheric conditions and electrical power supply.^29^ The gaseous carbon source (CH_4_) and silver bulk are decomposed/evaporated into energetic atoms/ions state (Fig. 5(a)), during which the ionized H^+^ can further promote the evaporation of Ag bulk through an energy exchange, and work as small carrier atoms. The subsequent stage goes into nucleation of Ag seeds, which also induces the formation of MWCNTs according to a vapor–liquid–solid (VLS) mechanism^30^ (Fig. 5(b) and (c)). In VLS growth of CNTs, the second phase of the Ag particles, referred to as catalyst, directs and confines the growth of CNTs on a specific orientation and within a confined area. An Ag catalyst forms a liquid droplet by itself or by alloying with the growth material of the C atoms during growth, and also acts as a trap for carbonic species. Enriched growth species in the catalyst droplets subsequently precipitate at the growth surface, resulting in one-directional growth. It has been predicted that the Ag can serve as a catalyst to grow CNTs, due to the surface of Ag nanoparticles having the highest adatom chemical potential, which will absorb the C atoms from the saturated vapor.^31^ For the fcc metal crystallites (for example, the coinage metals of Cu, Ag, and Au), it is confirmed that the (111) facets have the lowest energy; hence the lowest in adatom diffusion barriers, *e.g.* 0.20 eV for the (111) facets of Ag.^32^ This implies that a significant acceleration of C atoms through diffusion can be achieved on specific facets of Ag particles, which favors the growth of CNTs along the 〈111〉 direction and extends the sp^2^ carbon network involving hexagons and pentagons to form a crystallized graphite layer. The existence at the tip of MWCNTs or migration from the carbon cages make Ag particles separated from the well-defined carbon nanostructures (Fig. 5(d) and (e)). The isolated carbon cages can continuously grow by absorbing C atoms to form huge ones. The ultimately giant carbon fullerenes and the Ag nanoparticles are finally coated by thin layers of disordered carbon. It was reported earlier that the presence of Fe, Co, or Ni in the arc-discharge can stimulate the growth of the walled carbon tubes^33,34^ and strings of spherical beads. In this work, the same phenomena were also found; however, the carbon shell on the Ag nanocapsules was 1–2 nm in thickness, thinner than in the case of other metals.

### Electronic transport behaviors of Ag@C/MWCNT nanocomposite

**Fig. 6:**
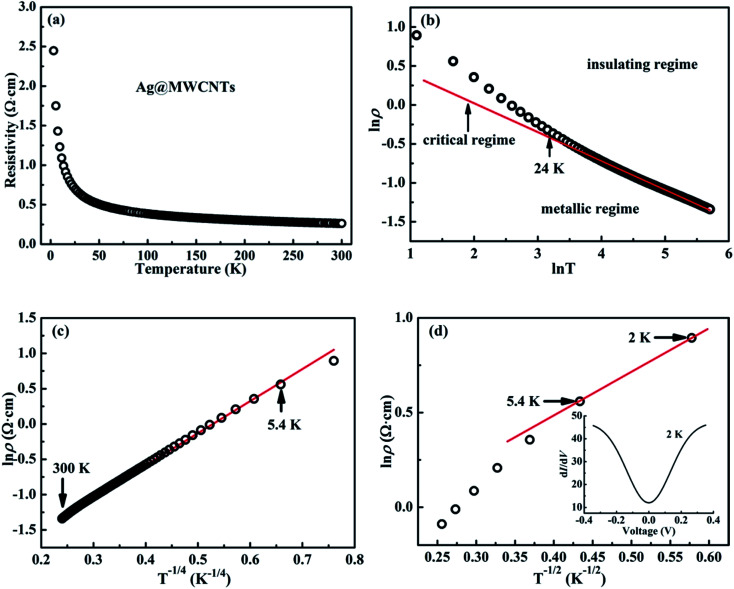
(a) Temperature-dependent resistivity of the Ag@C/MWCNT nanocomposite from 2 to 300 K; (b) ln–ln plot of the resistivity *ρ* as a function of the temperature *T* from 2 to 300 K; (c) ln *ρ vs. T*^−1/4^ plot fitted with MD VRH mode, satisfactorily fitted in the temperature range of 5.4–300 K; and (d) ln *ρ vs. T*^−1/2^ plot fitted with SE VRH mode, further confirmed an applicable temperature range of 2–5.4 K, and the inset shows plot of d*I*/d*V* as a function of *V* at 2 K.

Fig. 6 shows the temperature-related electronic transport of the mechanically compressed Ag@C/MWCNT sheet. It is demonstrated in Fig. 6(a) that the dielectric behavior is dominant in the temperature range of 2–300 K, while the similar behavior to pure CNTs suggests that the MWCNTs are the main electric contributors, although the metallic Ag@C NCs are also involved in the composite.^35^ The resistivity of the Ag@C/MWCNT nanocomposite increases with decreasing temperature, from 0.26 Ω cm (at 300 K) to 2.43 Ω cm (at 2 K), with a ratio *ρ*_r_[*ρ*_(2 K)_/*ρ*_(300 K)_] of approximately 9.35. The temperature-dependent resistivity of carbonaceous composite is usually classified into three regimes: metallic, critical, and insulating regimes, depending on the extent of disorder.^36^ These regimes are divided from the plot of ln *ρ vs.* ln *T*, as shown in Fig. 6(b), in which the transition temperature from metallic to insulating is determined as 24 K. This means that the Ag@C/MWCNT composite will preserve its metallic features down to this low temperature. The important factors affecting the conductivity of carbonic species are the ratio of sp^2^ to sp^3^ and the clustering of the sp^2^ sites, which significantly control the electronic properties of the carbon-based materials.^37^ The status of sp^2^ to sp^3^ bonds in Ag@C/MWCNT nanocomposite is well presented in the Raman spectrum (see Fig. 5), indicated by the intensities of the D and G bands. Consequently, the electrons in Ag@C/MWCNT composite are strongly localized, and its conductivity is probably described by hopping conduction mode, which is controlled by the hopping of electrons between local states near the Fermi level. When the Coulomb interaction of electrons is negligible, a three-dimensional Mott–David (MD) VRH^8^ is expected for temperature-dependent resistivity:1
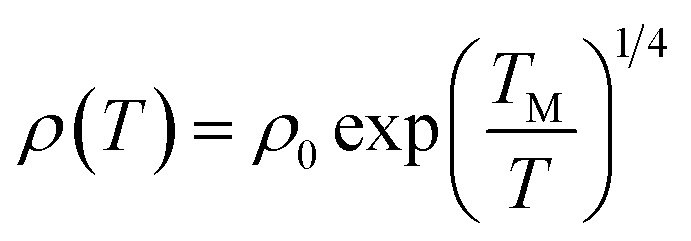

2 *T*_M_ = 512/9π*ξ*^3^*N*(*E*_F_)*k*_B_

where *T*_M_ is the characteristic Mott temperature obtainable from the plot of ln *ρ*(*T*) ∼*T*^−1/4^, *ξ* is the localization length, *k*_B_ is the Boltzmann constant, and *N*(*E*_F_) is the density of states at the Fermi level. A good linear fit is satisfactory with the experimental data, as represented in Fig. 6(c), indicating that the Ag@C/MWCNT nanocomposite follows a three-dimensional MD VRH model in the temperature range from 5.4 K to 300 K, with Mott temperature *T*_M_ = 440 K. Below 5.4 K, a slight deviation appears from the fitting line, which implies a transition of the transport behavior occurred from MD to Shklovskii–Efros (SE) VRH mode.^38^ Details are presented in SE mode, as shown in Fig. 6(d). The Coulomb interaction between the electrons becomes significant in the SE mode, and the resistivity can be described by following relationship:3
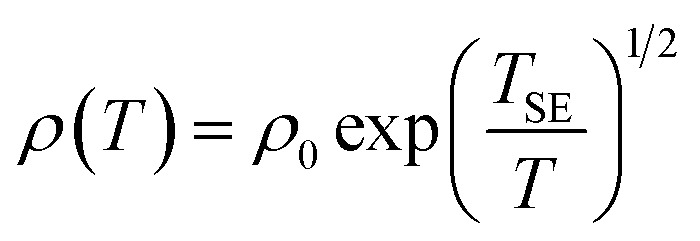

4 *T*_SE_ = 2.8*e*^2^/*εξk*_B_

where *ρ*_0_ is a material constant, *T*_SE_ is the characteristic SE temperature, and *ε* = *ε*_0_ + 4π*e*^2^*ξ*^2^*N*(*E*_F_) is the dielectric constant. From the slope of the ln *ρ*(*T*) ∼ *T*^−1/2^ line in Fig. 6(d), *T*_SE_ is calculated as 5.38 K. Such a transition of the transport behavior from the MD to the SE mode has also been observed in CNTs or carbon fiber materials.^35,39^

It is well known that a Coulomb interaction between electrons plays an important role in the transport behavior of a disordered system. An obvious suppression in differential conductance at lower bias voltages is shown in the inset of Fig. 6(d). The depletion of the density of the states at low energies is always regarded as a signature of Coulomb interaction between electrons, in accordance with the gradual opening of a Coulomb gap at low temperatures.^40^ Efros and Shklovskii^38^ have proposed that the Coulomb gap *Δ*_C_ can be obtained by the following relationship:

5 *Δ*_C_ = *e*^3^*N*(*E*_F_)^1/2^/*ε*^3/2^

From eqn (2) and (4), we have

6 *Δ*_C_ ≈ 0.905*k*_B_*T*_M_^−1/2^*T*_SE_^3/2^

From here, a Coulomb gap *Δ*_C_ ≈ 0.05 meV for the hybrid system is obtained. A transition point of electronic transport model has been reported at 15–16 K for the CNTs.^35,41^ Compared with above, the Ag@C/MWCNT composite has a much lower transition point, at 5.4 K (Fig. 6(d)). It might be reasonable that in the process of electron transport, silver nanoparticles can play the role of a bridge between the C–C; a large number of electrons can be free transport without being strongly localized. Therefore, the Coulomb interaction between electrons is weakened, so that the transition point of the transport model is backward delayed. It can be predicted that with increases in content of Ag nanoparticles, the model transition point will continue to be backward delayed, and eventually disappear.

## Conclusions

Ag@C/MWCNT nanocomposite was prepared *in situ* by a modified arc-discharge plasma approach, through evaporation of bulk silver in a methane (CH_4_) atmosphere. The Ag@C/MWCNT nanocomposite consists of carbon-coated Ag@C nanocapsules with a mean diameter of ∼10 nm, MWCNTs with about 17–32 graphite layers in the wall and a thickness in the range of 7–10 nm, and spherical carbon cages (giant fullerenes) with approximately 20–30 graphite layers in shell thickness. Temperature-dependent electrical resistivity reveals that the dielectric behavior is dominant in the Ag@C/MWCNT nanocomposite following MD VRH [ln *ρ*(*T*) ∼ *T*^−1/4^] mode and further transition into SE VRH [ln *ρ*(*T*) ∼ *T*^−1/2^] mode at 5.4 K. Such a transition is thought to be due to an enhanced Coulomb interaction caused by the modification from metallic Ag cores dispersed in the MWCNT matrix. The Coulomb gap, *Δ*_C_ ≈ 0.05 meV, is obtained for this nanocomposite system. Such an *in situ* fabricated Ag@C/MWCNT nanocomposite is expected to be a promising semiconductor for various applications in electronic devices.

## Conflicts of interest

There are no conflicts of interest to declare.

## Supplementary Material
